# Contribution of Aerobic Cellulolytic Gut Bacteria to Cellulose Digestion in Fifteen Coastal Grapsoid Crabs Underpins Potential for Mineralization of Mangrove Production

**DOI:** 10.1007/s00284-024-03718-5

**Published:** 2024-06-14

**Authors:** Cheuk Yan Lee, Shing Yip Lee

**Affiliations:** Simon F.S. Li Marine Science Laboratory, School of Life Sciences, The Chinese University of Hong Kong, Hong Kong SAR, China

## Abstract

Grapsoid crabs (Decapoda: Grapsoidea) inhabiting along the land-sea transition provided various amounts and quality of vascular plant carbon (e.g., fresh mangrove leaf, leaf litter, and mangrove-derived organic carbon) and perform differing levels of herbivory. Other than endogenous cellulase, symbiotic cellulolytic bacteria could also contribute to the crabs’ vascular plant carbon assimilation and mineralization. In this study, we isolated culturable cellulolytic bacteria from three gut regions (i.e., stomach, midgut, and hindgut) of 15 species of grapsoid crabs that inhabit in various coastal habitats (i.e., land margin, mangrove forest, tidal flat, and subtidal area). *Bacillus*, which was isolated from 11 out of the 15 grapsoid crabs, was the most common genus of culturable prominently cellulolytic bacteria among the target species. Seventy to ninety nine percent of culturable cellulolytic bacteria were removed, and the endoglucanase activity of five species was significantly reduced by 14.4–27.7% after antibiotic treatment. These results suggest that cellulolytic bacteria play a role in assisting mangrove carbon utilization in coastal grapsoid crabs, especially those inhabiting mangrove, mudflat, and subtidal areas. The significantly higher abundance of cellulolytic bacteria and the generally higher hydrolytic capacity of the bacteria in mangrove crab species suggest that they receive more contribution from symbionts for mangrove carbon utilization, while semi-terrestrial crabs seem to depend little on symbiotic cellulase due to the lower abundances.

**Supplementary Information:**

The online version contains supplementary material available at 10.1007/s00284-024-03718-5.

## Introduction

Mangroves are highly productive ecosystems, storing large amounts of carbon in the form of mangrove biomass and sediment organic matter [1]. The high C/N ratio, high content of structural carbon, and secondary metabolites [2, 3] of mangrove organic matter make fresh mangrove production generally unpalatable to macrofauna [2]. This organic matter was proposed to enter the food web through the detrital food chain, after enrichment by micro-organism such as bacteria and fungi [4]. Grapsoid crabs, especially mangrove sesarmid crabs, are one of the major processors of mangrove leaf litter [5, 6]. The ways by which these brachyuran crabs deal with mangrove organic matter as food is, however, unclear. Cellulase is one of the major tools used by macro-organisms to deal with their herbivorous or detritivorous diets [7]. Robust evidence exists that cellulase production is common among crustaceans [8, 9].

Partnership with symbionts for cellulose digestion is commonly adopted across the Tree of Life. Intestinal microorganisms play a role in important physiological functions such as digestion [10]. Multiple studies have reported the composition of symbionts of crabs using metagenomics [11, 12], with predicted functions of the symbionts presented without direct evidence from functional tests. Direct evidence of gut microbiota contributing to the nutrition of a sesarmid crab with stable isotope tracers was provided by Bui and Lee [13]. Many studies have reported cellulolytic symbionts in herbivorous or detritivorous organisms [14, 15], and it is possible that grapsoid crabs also possess symbiotic cellulases to assist in the assimilation of vascular plant material.

In the last decade, new cellulolytic bacteria from various sources such as the gut of various organisms [16], soil [17], and plants [18] have continually been reported. Most of these studies focus on the benefit of cleaner energy sources from cellulolytic bacteria, while few investigated their ecological roles. Cellulolytic bacteria are common in mangrove sediments [17, 19] and in mangrove trees as endophytes [18]. Therefore, it is possible that the crabs to acquire horizontally transmitted cellulolytic symbionts to facilitate cellulose digestion. To further explore the tools used by grapsoid crabs deal with mangrove organic matter, the cellulolytic symbionts of 15 grapsoid species along the land-sea transition in mangrove-lined coastal habitats were isolated and their potential contribution to host cellulase production was assessed in this study.

## Materials and Methods

### Crab Collection and Maintenance

Three replicates of male individuals of each crab species were collected for the isolation of symbiotic cellulolytic bacteria (Table 1). Crabs were collected from Mai Po Nature Reserve (22° 30′ N, 114° 02′ E), Ting Kok (22° 28′ N, 114° 13′ E), and Wu Shek Kok (22° 32′ N 114° 12′ E) in Hong Kong. As the physiology of female crabs may be significantly influenced by oogenesis, only male crabs were used to reduce unexplained individual variations. The crabs were maintained individually in cylindrical growth compartments (75 × 85 mm; diameter x height) with a small volume of seawater, freshwater, or brackish water added depending on the crab’s habitat, to prevent dehydration. Before dissection, the crabs were starved for two to four days depending on the time needed for evacuation of their guts.

**Table 1:** The grapsoid species studied

	Species	Field sites
Limnic	*Varuna yui*	TK
Semi-land	*Chiromantes haematocheir*	
	*Chasmagnathus convexus*	
	*Neosarmatium indicum*	
	*Parasesarma pictum*	
	*Orisarma dehaani*	
	*Orisarma intermedium*	
	*Orisarma patshuni*	
Mangrove	*Parasesarma bidens*	
	*Episesarma versicolor*	WSK
	*Parasesarma affine*	MP
Mudflat/Sandflat	*Metaplax longipes*	TK
	*Metopograpsus frontalis*	
Shallow subtidal region	*Gaetice depressus*	
	*Hemigrapsus penicillatus*	

*TK* Ting Kok, *WSK* Wu Shek Kok, *MP* Mai Po nature reserve

### Isolation of Cellulolytic Bacteria

The stomach, midgut, and hindgut of the crabs were dissected out, homogenized, and vortexed in 500 µl phosphate-buffered saline (PBS) solution. To obtain isolated colonies, the mixture was then serially diluted (1:100, 1:1000, and 1:10,000) and the bacteria present were cultured on CMC-agar consisted of carboxymethyl cellulose (CMC) (Aldrich), 5; yeast, 0.5; (NH_4_)_2_SO_4_, 1; KH_2_PO_4_, 0.5; KCl, 0.1; MgSO_4_, 0.25; and agar, 1.5 (all in g per 0.5 L), following the method of Thomas et al. [20]. The number of colonies was counted, and the abundance of bacteria was calculated. 0.5% Congo-red and sodium chloride were used as indicators for cellulose degradation in CMC-agar to identify cellulolytic bacteria [21]. A clear zone around colonies indicated cellulose degradation. The cellulolytic bacteria were further investigated. The average hydrolytic capacity (HC) (clear zone diameter/colony diameter) of bacteria of each genus in each gut region was calculated.

### Bacteria Identification and Phylogenetic Analysis

Prominently cellulolytic bacteria with HC higher than one were further identified by sequencing their 16S rRNA genes. These bacteria were picked for 16S rRNA gene amplification through colony PCR [22] in 50 µl reaction with primers 27F (5′-AGAGTTTGATCMTGGCTCAG-3′) and 1492R (5′-GGTTACCTTGTTACGACTT-3′) using the following profile: an initial denaturation at 95 °C for 3 min; 35 cycles of thermal cycling, each consisted of a denaturation step at 95 °C for 30 s, an annealing step at 55 °C for 30 s and an extension step at 72 °C for 1 min; final extension at 72 °C for 10 min, and holding at 4 °C. The PCR products were sent to BGI (Hong Kong) for quality check and Sanger sequencing. Only sequences with Phred quality scores ≥ 30 were analyzed (see Supplementary file for details on quality assurance). In order to identify the bacteria isolated, their nearly full-length16S rRNA gene sequences (~ 1300 bp) were analyzed using Nucleotide Blast algorithm within the website of the National Center for Biotechnology information (http://blast.st-va.ncbi.nlm.nih.gov/Blast.cgi). The sequences were blasted against the database of 16S ribosomal RNA sequences (Bacteria and Archaea). The identity of bacteria was determined when the BLAST high hit reaches E-value = 0 and identity ≥ 98%. In order to elucidate their evolutionary relationship with their congeners, the 16S rRNA gene sequences obtained were used to plot phylogenetic trees together with 16S rRNA sequences retrieved from NCBI. Sequences were aligned using MUSCLE [23]. The substitution model and phylogenetic tree were chosen and built by using RAxML [24].

### Data Analysis

Two-way ANOVA was used to test the effect of habitat and crab taxa on the abundance of CMC-agar-screened cellulolytic bacteria. Significant results of ANOVA were further analyzed by applying Tukey’s Honestly Significant Difference (HSD) post hoc test to detect differences among groups.

### Symbiotic Cellulase Contribution in the Host Stomach

In this experiment, 10 replicates of male individuals were examined for each crab species. To estimate the bacterial contribution to cellulase production in the crabs, endoglucanase activity in the gastric juice was measured before and after the suppression of bacteria. The stomach was chosen for the estimation because we can monitor the same individual both before and after the treatment. The decrease in endoglucanase activity was taken as the bacterial cellulase contribution. Antibiotic–Antimycotic agent (ThermoFisher) was used to suppress bacteria activity and, thus, enzymatic contribution, in the gut of crabs. The antibiotic was diluted and applied to the water at 1 × concentration in the crabs’ containers. The crabs were treated with antibiotics for 24 h. The crabs were then euthanized by placing them in ice slurry, and their gastric juice extracted using a clean syringe with a blunt-ended needle. Endoglucanase activity was determined as the rate of production of reducing sugars from carboxymethyl cellulose (CMC). 5 μl of gastric juice was added to 45 μl 2% CMC (Sigma) and incubated for 10 min at 37 °C with 300 rpm agitation. The mixture was transferred to ice slurry to terminate the reaction before the measurement of reducing sugars produced using the tetrazolium blue method [25]. Control sets (*n* = 10) for the measurement of endoglucanase activity change in 24 h without antibiotics treatment were also performed.

### Data Availability

The 16S rRNA sequences of isolated cellulolytic bacteria were submitted to Genbank repository (Accession no.: OP393481–OP39386).

## Results

### Abundance of Gut Bacteria

**Fig. 1:**
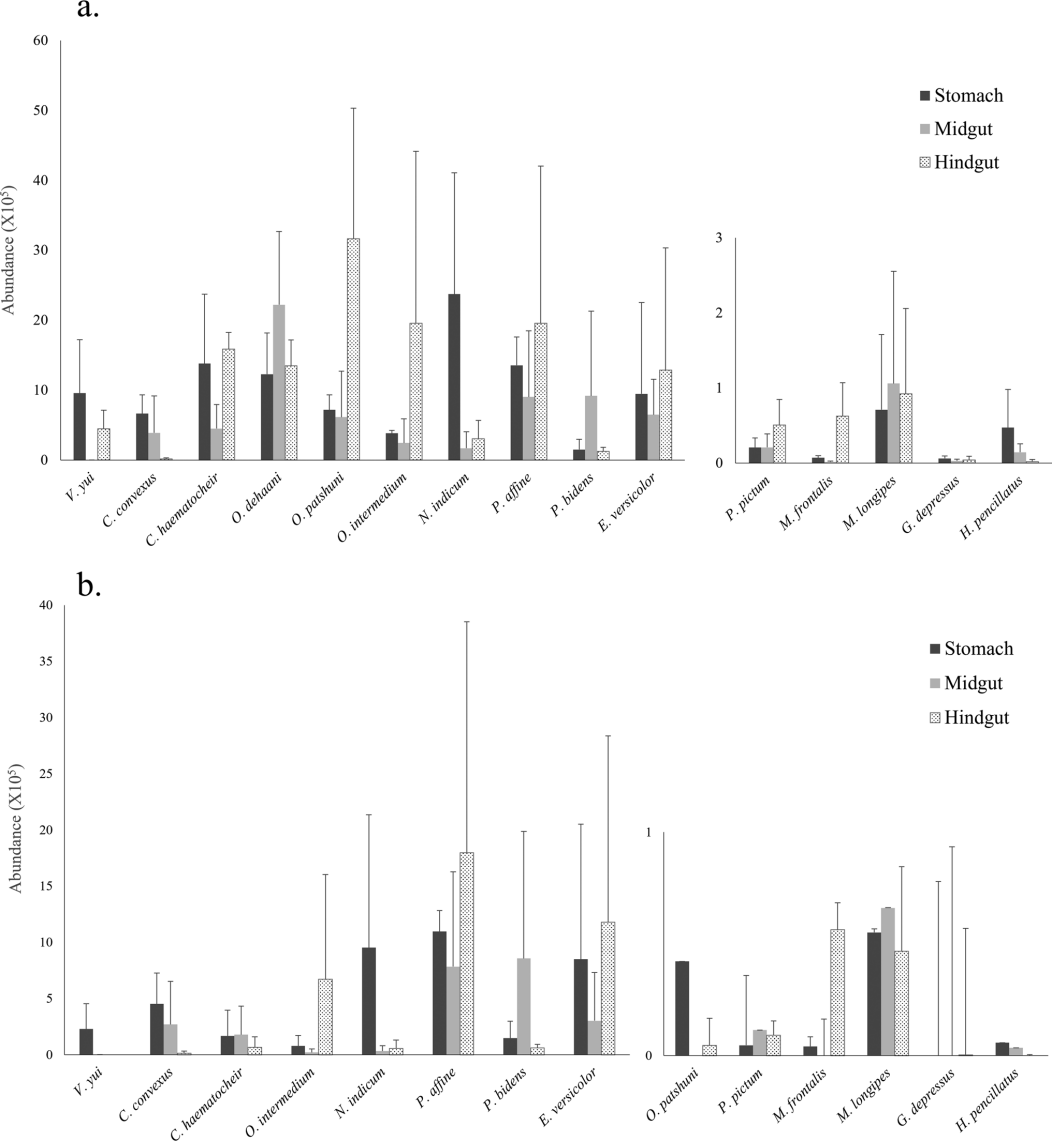
**a** Mean (+ SD) abundance of bacteria isolated by CMC-agar (*n* = 3 for each species). **b** Mean (+ SD) abundance of cellulolytic bacteria (*n* = 3 for each species). There is great intraspecific and interspecific differences in the abundance of isolated bacteria. However, there is statistically significant differences (Tables 2 and 3) in the abundance of isolated cellulolytic bacteria between crabs from different families and habitats, suggesting that crabs might change strategies for plant materials digestion according to taxa and habitats

Both aerobic and facultative anaerobic cultural bacteria were isolated. The Congo-red dye test showed that not all the bacteria selected by CMC-agar were cellulolytic. There was large interspecific and intraspecific variation in the abundance of both CMC-screened bacteria and cellulolytic bacteria (Fig. 1). There was no particular pattern of bacteria abundance in the different gut regions. In general, the abundance of CMC-screened and cellulolytic bacteria in subtidal and mudflat species was much lower than that of crabs from the other regions (Fig. 1). The land crabs *Orisarma dehaani* and *O. patshuni* also showed lower cellulolytic bacteria abundances compared to the mangrove and other land species (Fig. 1b). Two-way ANOVA showed that the abundance of CMC-screened bacteria in sesarmids was significantly higher than those of varunids and grapsids. Abundance in crabs from the land region also was significantly higher than those in the mudflat and subtidal species (Table 2). Two-way ANOVA results also showed that habitat had a significant effect on the abundance of cellulolytic bacteria, but taxa did not. The abundance of gut cellulolytic bacteria of crabs in the mangrove was significantly higher than those of the limnic, semi-terrestrial, mudflat, and subtidal species (Table 3). Semi-terrestrial crabs showed similar levels of total bacteria abundance but significantly lower abundance of cellulolytic bacteria when compared to those associated with mangrove species (Table 3). For example, there was no sign of cellulolytic bacteria in the gut of *O. dehaani*. In general, the occurrence of prominently cellulolytic bacteria in land and subtidal crabs was patchy while those in mangrove were more frequent (e.g., Fig. S1).

**Table 2:** Comparison of the abundance of CMC-screened bacteria in crabs from different families/habitats

Family/habitat (I)	Family/habitat (II)	Mean difference in abundance of CMC-screened bacteria (I–II) (μmol min^−1^ ml^−1^)
Sesarmid	Varunid	8.65 ± 2.03 × 10^5^***
	Grapsid	1.03 ± 3.84 × 10^6^*
Land	Mudflat	9.53 ± 2.92 × 10^5^*
	Subtidal	9.97 ± 2.92 × 10^5^**

*P ≤ 0.05, **P ≤ 0.01, ***P ≤ 0.001, denoting significant differences in abundance of CMC-screened bacteria

**Table 3:** Comparison of the abundance of cellulolytic bacteria in crabs from different families/habitats

Habitat (I)	Habitat (II)	Mean difference in the abundance of cellulolytic bacteria (I–II) (μmol min^−1^ ml^−1^)
Mangrove	Limnic	7.10 ± 2.43 × 10^5^*
	Land	6.41 ± 1.46 × 10^5^***
	Mudflat	7.48 ± 1.92 × 10^5^**
	Subtidal	7.85 ± 1.92 × 10^5^**

*P ≤ 0.05, **P ≤ 0.01, ***P ≤ 0.001, denoting significant differences in abundance of cellulolytic bacteria

### Prominently Cellulolytic Symbionts and Their Hydrolytic Capacity

Bacteria with hydrolytic capacity (HC) larger than one were counted as prominently cellulolytic (PC) bacteria in this study. Bacteria were identified by BLASTn analysis of the 16S rRNA gene against the NCBI database. The 16S rRNA gene sequences generated in this study can be retrieved from NCBI database (Accession number please refer to Supplementary file 1). Thirteen genera of PC bacteria were identified (Table 4). *O. dehaani* was the only species with no cellulolytic bacteria isolated. *Bacillus* was the most common genus, which was isolated from 11 species. A particularly higher diversity of PC bacteria with usually more than three genera was observed in semi-terrestrial crabs. For instance, six and five genera of PC bacteria were isolated from *Chasmagnathus convexus* (Accession no.: OP393783–OP393796) and *Chiromantes haematocheir* (Accession no.: OP393595–OP393620), respectively. Only one or two genera were identified in host species from the other environments. In general, dominant genus of PC bacteria could be identified in the limnic, mangrove, and mudflat species. *Bacillus* was the dominant PC cellulolytic genus in *Parasesarma affine* (Accession no.: OP393481–OP393503), *P. bidens* (Accession no.: OP393504–OP393536) and *Metopograpsus frontalis* (Accession no.: OP393670–OP393710) along their entire gut. *Mangrovibacter* and *Vibrio* were the dominant genera in *Episesarma versicolor* (Accession no.: OP393567–OP393584) and *Varuna yui* (Accession no.: OP393832–OP393866), respectively. PC *Vibrio*, *Mangrovibacter,* and *Microbacterium* were shared by more than one crab species*.* There were exclusive genera of PC bacteria isolated from *C. haematocheir*, *C. convexus*, *Orisarma intermedium*, *Neosarmatium indicum*, *Parasesarma pictum*, *E. versicolor,* and *M. frontalis*. PC bacteria were present in all the three regions of the gut of mangrove crabs, while there was often the absence of PC bacteria in the midgut of semi-terrestrial crabs.

**Table 4:** The hydrolytic capacity of prominently cellulolytic bacteria isolated from the gut of crabs along the land-sea transition

		Stomach	Midgut	Hindgut
Limnic	*Varuna yui*	***Vibrio*** (2.6) {33.3%}	***Bacillus*** (1.8) {33.3%}	***Bacillus*** (1.8) {33.3%}
Land	*Chiromantes haematocheir*	[*Novosphingobium*] (3.4) {17.5%}	[*Novosphingobium*] (3.7) {30.1%}	[*Novosphingobium*] (4.2) {20%}
		*Cellvibrio* (4.6) {16.7}	*Bacillus* (3.8) {3.3%}	*Bacillus* (4) {13.3%}
		*Microbacterium* (3.9) {16.7%}		
	*Chasmagnathus convexus*	[***Pseudocitrobacter***] (1.5) {66.7%}	[***Pseudocitrobacter***] (1.5) {33.3%}	[***Pseudocitrobacter***] (1.6) {66.7%}
		[*Demequina*] (4.7) {7.4%}		
		[*Burkholderia*] (2) {14.8%}		
		[*Novosphingobium*] (2.4) {7.4%}		
		*Microbacterium* (3) {3.7%}		
	*Orisarma dehaani*	/	/	/
	*Orisarma intermedium*	[***Klebsiella***] (2.5) {66.7%}		[***Klebsiella***] (2.4) {33.3%}
	*Orisarma patshuni*	*Microbacterium* (2) {11.1%}		
		*Bacillus* (2) {6.7%}		
		*Vibrio* (5) {22.2%}		
	*Neosarmatium indicum*	[*Erythrobacter*] (5) {22.2%}	[*Ramlibacter*] (2.5) {16.7%}	[*Actinobacterium*] (2.3) {20%}
		[*Humibacillus*] (3.1) {11.1%}		[*Isoptericola*] (3) {6.7%}
		*Mangrovibacter* (5) {33.3%}		*Mangrovibacter* (3.5) {33.3%}
	*Parasesarma pictum*	*Bacillus* (3.7) {66.7%}		*Bacillus* (2.6) {30.7%}
				*Microbacterium* (3.1) {33.3%}
				[*Brevibacillus*] (3.2) {2.6%}
Mangrove	*Parasesarma bidens*	***Bacillus*** (8.6) {50%}	***Bacillus*** (5) {50%}	***Bacillus*** (9.5) {51.1%}
	*Parasesarma affine*	***Bacillus*** (2.9) {100%}	***Bacillus*** (4) {100%}	***Bacillus*** (4.6) {100%}
	*Episesarma versicolor*	***Bacillus*** (5) {7.9%}	***Bacillus*** (2) {18.5%}	*Microbacterium* (1.9) (33.3%)
		[*Lysinibacillus*] (9) {25.9%}	***Mangrovibacter*** (2) {14.8%}	*Mangrovibacter* (4.5) {28.4%}
				*Bacillus* (2.1) {4.9%}
Mudflat	*Metopograpsus frontalis*	***Bacillus*** (1.8) {98.6%}		***Bacillus*** (3.2) {100%}
		[*Paenibacillus*] (3.7) {1.2%}		
	*Metaplax longipes*	*Bacillus* (1.35) {33.3%}	*Bacillus* (1.4) {33.3%}	*Bacillus* (1.3) {33.3%}
Subtidal	*Gaetice depressus*			*Bacillus* (2.2) {100%}
	*Hemigrapsus penicillatus*	***Bacillus*** (7.1) {25%}	***Bacillus*** (6.6) {33.3%}	***Bacillus*** (7.5) {21.2%}
		*Vibrio* (4) {33.3%}		

Bold: Dominant cellulolytic bacteria; ( ): Hydrolytic capacity; [ ]: Prominently cellulolytic bacteria exclusively isolated from the species. { }: The average relative contribution to the abundance of prominently cellulolytic bacteria

The HC of PC bacteria ranged from 1.3 to 9.5 (Table 4). The highest HC was recorded from *Bacillus* (*P. bidens*—hindgut) and the lowest were also *Bacillus* (*M. longipes*—hindgut). Semi-terrestrial *Chiromantes haematocheir,* subtidal *H. penicillatus,* and all mangrove crabs had *Bacillus* with HC ≥ 4 isolated from at least one region of their guts. In contrast, the gut of the other species had *Bacillus* with HC around 2. The HC of bacteria from the mangrove crabs was generally higher than those of crabs from the other environments.

### Contribution of Symbiotic Cellulase in Host Stomach

Between 70 and 99% of CMC-screened bacteria were eliminated from the stomach of the crabs after the 24 h antibiotics treatment. All crab species except *O. dehaani* had their endoglucanase activity in gastric juice reduced after the 24 h antibiotics treatment. Five species showed a significant decrease (14.4 to 27.7%) (Table 5) while endoglucanase activity in *O. dehaani* increased significantly.

**Table 5:** Decrease in endoglucanase activity in crabs compared with the control group after the 24 h antibiotics treatment

Species	Decrease in endoglucanase activity after antibiotics treatment (%)
*Parasesarma pictum*	14.4***
*Parasesarma bidens*	16.5*
*Metaplax longipes*	20.0**
*Metopograpsus frontalis*	14.6*
*Hemigrapsus penicillatus*	27.7*

Activity in *G. depressus* also decreased significantly after antibiotics treatment, but the control group of this species also had a significant decrease, which canceled out the effect of antibiotics

*P ≤ 0.05, **P ≤ 0.01, ***P ≤ 0.001

### Phylogenetic Analysis

**Fig. 2:**
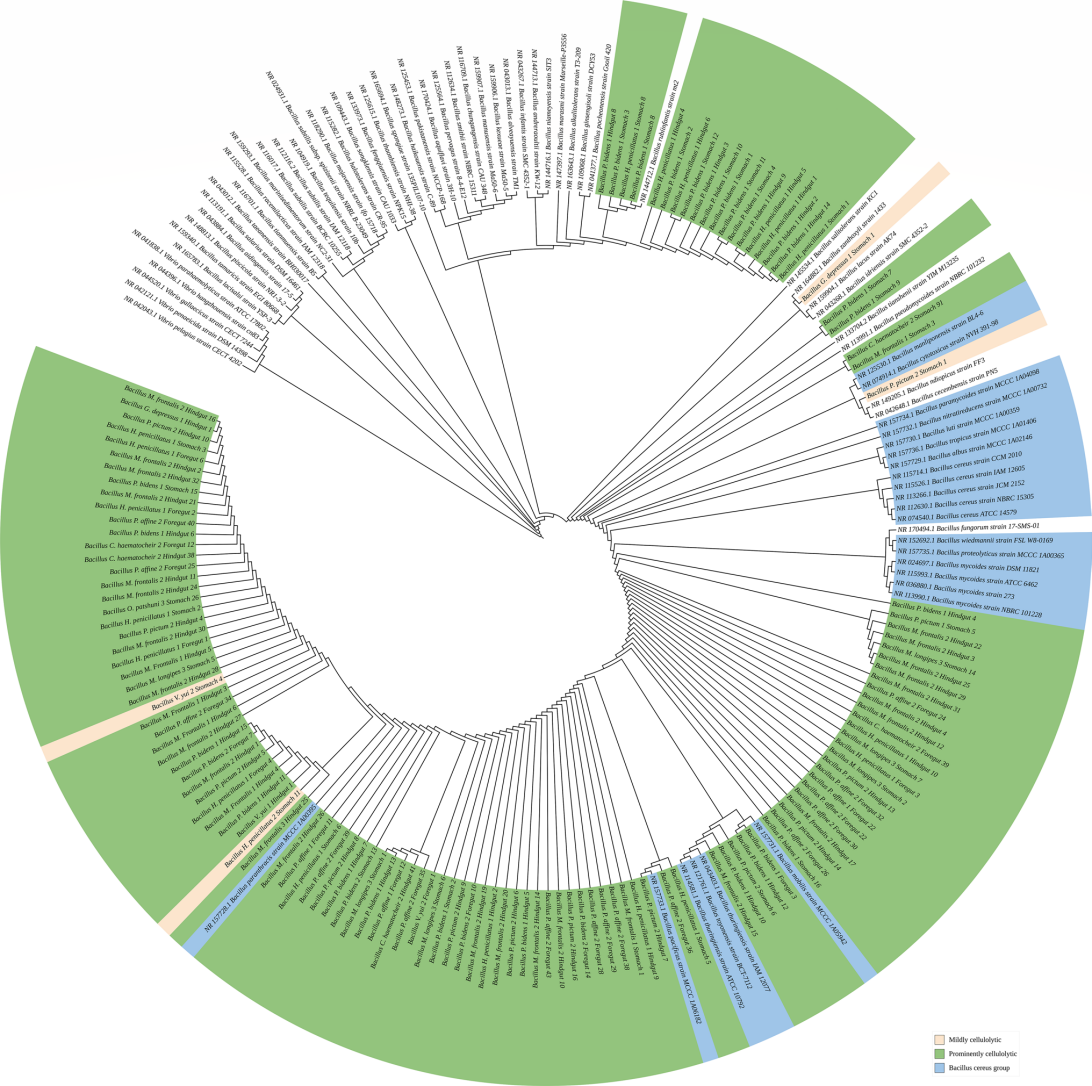
The phylogenetic tree of *Bacillus. Bacillus* was isolated from 11 out of the 15 grapsoid crabs. Some of them are closely related to *Bacillus cereus* group, which occurs in marine or coastal environments, suggesting the possibility that isolated Bacillus was acquired by the crabs from their habitats

The model selection (Table S1) and the phylogenetic tree plotting were done by RAxML. In each phylogenetic tree, there is no distinct clade identifiable according to habitats, host taxa, or gut regions. *Bacillus*, *Vibrio*, *Klebsiella*, *Mangrovibacter,* and *Microbacterium* showed higher levels of species diversity, the bacteria isolated clustered with different species (Figs. 2, S2–S6), while *Pseudocitrobacter* isolated seems to be very closely related (Fig. S6). The majority of *Bacillus* isolated seem to be closely related to the *Bacillus cereus* group (Fig. 2). Mildly cellulolytic (HC = 1) and PC *Klebsiella* are clustered into two different clades (Fig. S3).

## Discussion

Different grapsoid crabs host different combinations of PC bacteria (Table 4). The inconsistency of PC bacteria species isolated from the replicates of each host suggests that the bacteria are likely to be horizontally instead of vertically transmitted. The majority of *Bacillus* isolated seemed to be closely related to the *Bacillus cereus* group (Fig. 2), which is a group of *Bacillus* from marine environments [26] and ubiquitous in soil and mangrove environment [19, 27]. This explains the widespread of *Bacillus* sp. among the crabs (Table 4). *Vibrio*, *Klebsiella*, *Mangrovibacter,* and *Microbacterium* are also commonly found in marine environments such as the water column, nearshore, or mangrove sediments [28–32]. These bacteria include *Vibrio hispanicus*, *Klebsiella pneumoniae*, *Microbacterium awajiense*, *M. fluvii,* and *M. mangrovi*, which showed a close relationship with the bacteria isolated in this study (Figs. S2, S3 and S5). It is highly possible that the grapsoid crabs acquired their respective associated cellulolytic bacteria from their habitats. The bacteria could be transported through the water body or tidal flow, which might explain (1) the presence of *Vibrio* in limnic (*Varuna yui*) and subtidal (*Hemigrapsus penicillatus*) species (Fig. S2); and (2) the noticeably different PC bacterial community observed in grapsoid crabs from the land margin, which is farthest away from the sea.

The possession of cell-wall degrading enzymes (e.g., cellulase) is one of the traits of endophytes [33]. Predominantly herbivorous or detritivorous crabs in the land and mangrove regions probably ingest cellulolytic endophytes with their leaf intake as mangroves support diverse endophytes [34]. For instance, the endophytic bacteria isolated from leaves of the mangrove *Avicennia marina* include *Bacillus*, *Vibrio*, *Microbacterium*, *Citrobacter*, and *Lysinibacillus,* which match those isolated from grapsoid crabs along the land-sea transition (Table 4). On the other hand, *Vibrio plantisponsor* and *Mangrovibacter plantisponsor*, which are closely related to some of the *Vibrio* and *Mangrovibacter* isolated from grapsoid crabs, were isolated from the root of mangrove-associated wild rice, *Porteresia coarctata* [35, 36]. It was indicated that *Mangrovibacter* is an endophyte in the root of *Phragmites karka* [37]*,* which occurs in the local coastal wetlands. Therefore, crabs could also ingest cellulolytic endophytes through the consumption of plant roots. Further investigation of the environmental and endophytic bacterial communities is needed to ascertain the origin of gut cellulolytic bacteria in these grapsoid crabs.

Although there is great variation in bacteria abundance among species, significantly higher abundances of PC bacteria in the mangrove crabs were observed. This result was expected as plant materials contribute more to the diet of mangrove crabs than those from other habitats [38]. This high abundance may be the result of several uptake pathways. First, the crabs may ingest large numbers of cellulolytic bacteria from the mangrove sediment which is a common component of their gut contents. [38]. The ubiquity of *Bacillus* in mangrove sediment may explain its dominance among cellulolytic bacteria in *P. bidens* and *P. affine*. Second, mangrove crabs may acquire the PC endophytes associated with mangrove trees. *Bacillus*, which is the dominant PC bacteria genus in the mangrove crabs *P. bidens* and *P. affine*, is one of the common endophytes isolated from mangroves [18, 39]. The consumption of mangrove leaf litter by *P. bidens* [38] and *P. affine* [40] may result in the dominance of *Bacillus*. The herbivory or detritivory of these mangrove crabs may then facilitate the growth of cellulolytic bacteria and results in the high abundances of PC bacteria in their gut.

In general, stomach cellulase activity decreased after antibiotics treatment. Significant reductions (14.4% to 27.7%) were observed in five crab species (Table 5), including one semi-terrestrial, one mangrove, two mudflat, and one subtidal crabs. This is evidence that symbionts assist the digestion of cellulose in vascular plant or mangroves at least in certain grapsoid crabs. However, the results may not fully reflect the PC bacteria abundance in the crabs as the percentage of contribution from bacteria is also affected by the endogenous cellulase production of host and the cellulolytic level of the bacteria. For example, PC bacteria abundance was significantly higher in mangrove species than in subtidal species, but bacteria showed a higher contribution (27.7%) to gastric cellulase production in subtidal (*Hemigrapsus penicillatus*) than in mangrove species (*P. bidens,* 16.5%). The probably higher endogenous cellulase production of the mangrove species could eclipsed the contribution of gut bacteria.

Both semi-terrestrial and mangrove crabs perform high levels of herbivory or detritivory and have comparable endoglucanase activity in their gastric juice [41], but the mangrove species supporting significantly higher PC bacteria abundances (6.41 ± 1.46 × 10^5^) than in the semi-terrestrial crabs (Table 3). This result implies that semi-terrestrial crabs acquire little assistance from symbionts whereas mangrove species enjoy higher levels of symbiotic contribution. The significantly higher β-glucosidase (BGLU) activity in semi-terrestrial species might already facilitate sufficient acquisition of carbon from cellulose [41] and explain why symbiosis with cellulolytic bacteria may not be necessary and, therefore, not well developed among semi-terrestrial crabs. Among all grapsoids examined in this study, the semi-terrestrial *O. dehaani* showed a distinct difference in the results of both cellulolytic bacteria isolation and antibiotics treatment compared to the other species. No cellulolytic bacteria were isolated from *O. dehaani,* and it was the only species that showed increased endoglucanase activity after the 24 h antibiotics treatment. *O. dehaani* might solely depend on endogenous cellulases for cellulose digestion. It is also possible that pathogenic bacteria were removed by antibiotics and more cellulase was, therefore, produced by *O. dehaani* under improved health condition. The lack of a significant effect of antibiotic treatment on the cellulase production in other crabs implies the limited contribution of cellulase production by bacteria in their stomachs. However, it is highly possible that symbionts also play a role in cellulase production in the midgut and hindgut where high abundances of them were found in some species (Fig. 1b). Further investigations on fermentation by obligatory anaerobic symbionts and the symbiotic cellulase production in the midgut and hindgut are needed to provide a thorough understanding of the role of cellulolytic symbionts in vascular plant detritus utilization and mangrove carbon mineralization of host.

PC *Pseudocitrobacter* sp. isolated from *Chasmagnathus convexus* had the highest resemblance to *Pseudocitrobacter anthropi* strain MP-4, which is a lignin-degrading bacterium isolated from termite, *Microtermes pakistanicus* [42]. If *Pseudocitrobacter* isolated in this study also possesses the same function, it could assist the digestion of both cellulose and lignin in plant materials in *C. convexus*. On the other hand, cellulolytic nitrogen-fixing bacteria have also been recorded, e.g., *Bacillus* [43] and *Cellvibrio* [44, 45]. Cellulolytic nitrogen-fixing bacteria have been isolated from shipworms [46]. If the cellulolytic bacteria isolated from grapsoids are also nitrogen fixing, the disadvantage of herbivory (high C/N ratio of the food) may be offset by the contribution from these bacteria. Recent data suggest that *Parasesarma bidens* meets some of its N needs through N-fixation, probably through the activities of soil or gut bacteria [47]. Further functional tests are needed to confirm the nitrogen-fixing role of these symbiotic cellulolytic bacteria.

Early views of the fate of mangrove production suggested that leaf litter mainly entered the coastal food web as detritus [4], with microorganisms assumed to be the initial processors of the litter. Previous studies focused on the chemical changes of mangrove leaf litter upon decomposition, e.g., [40, 48, 49] and the consumption and utilization of leaf litter by detritivores [5, 8], but not the mechanisms of how symbiotic bacteria might facilitate decomposition or mangrove carbon mineralization. The presence of cellulolytic symbionts in the digestive tract of grapsoid crabs, especially in the mangrove crabs, suggests that bacteria could promote mangrove carbon mineralization through their role as symbionts, but not just as decomposers in the environment. This study demonstrates that a significant part of the carbon mineralising capacity of grapsoid crabs actually comes from their symbionts.

The possible role of bacteria in other aspects of mangrove carbon mineralization has also been overlooked. For example, tannins are common in mangroves and could hinder the digestion of proteins. Symbiotic tannin-degrading cellulolytic bacteria including *Bacillus* have been isolated from herbivorous animals [50]. If such bacteria are also present in the gut of grapsoid crabs, the digestion of vascular plant materials with high tannin contents will be promoted through their removal by these bacteria. Symbiotic bacteria could have many critical roles in mangrove carbon mineralization, many of which are yet to be fully explored.

## Conclusion

The isolation of prominently cellulolytic (PC) bacteria and the results of the antibiotics treatment experiment demonstrate (1) the assistance of PC symbionts provide toward host cellulase production; and (2) their significant involvement in mangrove carbon mineralization. While the origin of this symbiotic association is yet to be further ascertained, the persistent and widespread occurrence of the bacteria in the guts of coastal grapsoid crabs points to a significant ecological role of these symbionts in the mineralization of mangrove production. The abundance of PC bacteria is significantly, and bacterial hydrolytic capacity generally, higher in mangrove species, suggesting that they receive more help from symbionts for mangrove carbon utilization. Semi-terrestrial crabs seem to depend little on symbiotic cellulases due to their lower abundances compared to endogenous cellulase. PC symbionts might be acquired from the environment or the diet of the crabs. Isolated from 11 out of 15 crab species, *Bacillus* was the most common genus of PC symbiont recorded in this study. It is likely that high abundances of PC symbionts also contribute to cellulase production in the midgut and hindgut of mangrove crab species. Therefore, PC bacteria probably contribute to cellulose digestion significantly more than previously reported. The physiological versatility of bacteria might allow them to participate in mangrove carbon mineralization in diverse ways.

### Supplementary Information

Below is the link to the electronic supplementary material.

Supplementary file1 (TIFF 7901 kb). Congo-red stained CMC-agar of bacteria isolated from the stomach of a *Chiromantes haematocheir*; and **b** *Parasesarma affine*.
The figure showed some examples of the cellulolytic bacteria isolated from *Chiromantes haematocheir* and *Parasesarma affine*. The cellulolytic bacteria were identified through the halo around the colonies.

Supplementary file2 (TIFF 13464 kb). The phylogenetic tree of *Vibrio*.
The *Vibrio* spp. isolated form a monophyletic clay with several species of *Vibrio*, therefore, it is not possible to narrow down the species at this point. It seems *Hemigrapsus penicillatus, Varuna yui, Orisarma patshuni* and *Parasesarma pictum* share similar Vibrio as there is no grouping of *Vibrio* spp. corresponding to the species of hosts.

Supplementary file3 (TIFF 11634 kb). The phylogenetic tree of *Klebsiella*.
Mildly cellulolytic and prominently cellulolytic *Klebsiella* spp. grouped into two different monophyletic clay in the 16s rRNA phylogenetic tree. According to the monophyletic grouping, prominently cellulolytic *Klebsiella* spp. isolated are quite likely to be certain strains of the *K. quasivariicola* and *K. variicola* while mildly cellulolytic *Klebsiella* spp. isolated are quite likely to be strains of *K. aerogenes*.

Supplementary file4 (TIFF 5992 kb). The phylogenetic tree of *Mangrovibacter*.
All the *Mangrovibacter* isolated are prominently cellulolytic and they were isolated from semi-terrestrial detritovorous *Episesarma versicolor* and Neosarmatium indicum. It is difficult to determine the species as there is far less studies about the genus when compared to other groups such as *Bacillus*.

Supplementary file5 (TIFF 15748 kb). The phylogenetic tree of *Microbacterium*.
*Microbacterium* spp. could be restricted to landward environment as it was only isolated from semi-terrestrial species. It seems there are various species isolated as they grouped to different clay with different species.

Supplementary file6 (TIFF 4689 kb). The phylogenetic tree of *Pseudocitrobacter*.
*Pseudocitrobacter* was only isolated from *Chasmagnathus convexus*. Although the *Pseudocitrobacter* isolated grouped monophyletically with two *P. anthropic* strains. The species itself is not monophyletic, therefore, the ones isolated cannot be determined as the same species.

Supplementary file7 (DOCX 28 kb)

## Data Availability

The datasets generated during and/or analyzed during the current study are available from the corresponding author on reasonable request, while 16S rRNA data generated are available in the Genbank repository, please refer to Supplementary file 1.
