# Supplementary figures and images for: Contribution of Aerobic Cellulolytic Gut Bacteria to Cellulose Digestion in Fifteen Coastal Grapsoid Crabs Underpins Potential for Mineralization of Mangrove Production
Source: Curr Microbiol. 2024 Jun 14;81(8):224. doi: 10.1007/s00284-024-03718-5 (PMC11178586; doi:10.1007/s00284-024-03718-5)

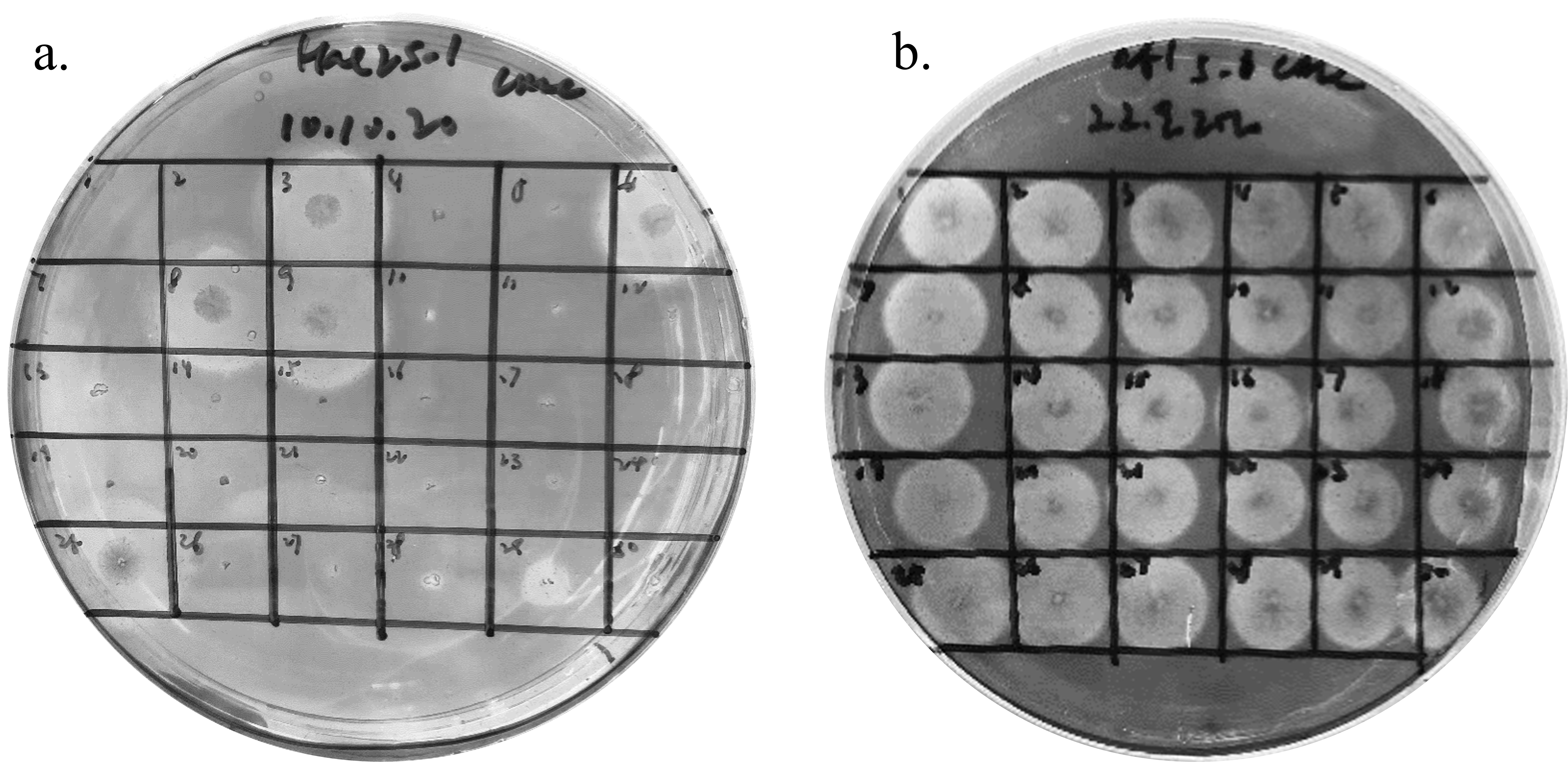

Supplement: Supplementary file 1 — Supplementary file1 (TIFF 7901 kb). Congo-red stained CMC-agar of bacteria isolated from the stomach of a Chiromantes haematocheir; and b Parasesarma affine. The figure showed some examples of the cellulolytic bacteria isolated from Chiromantes haematocheir and Parasesarma affine. The cellulolytic bacteria were identified through the halo around the colonies. [file 284_2024_3718_MOESM1_ESM.tiff]

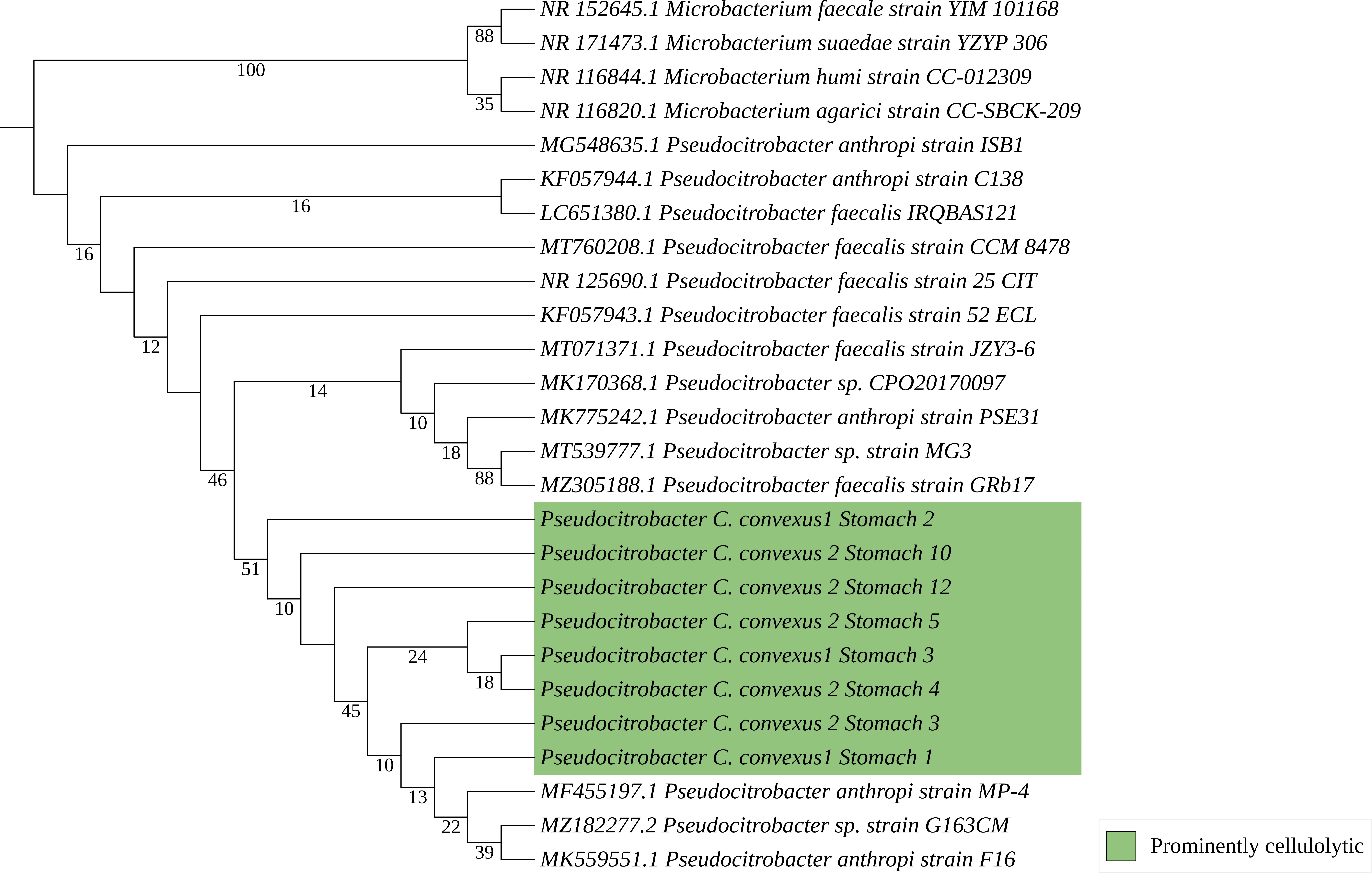

Supplement: Supplementary file 6 — Supplementary file6 (TIFF 4689 kb). The phylogenetic tree of Pseudocitrobacter. Pseudocitrobacter was only isolated from Chasmagnathus convexus. Although the Pseudocitrobacter isolated grouped monophyletically with two P. anthropic strains. The species itself is not monophyletic, therefore, the ones isolated cannot be determined as the same species. [file 284_2024_3718_MOESM6_ESM.tiff]
