# Supplementary material for: Contribution of Aerobic Cellulolytic Gut Bacteria to Cellulose Digestion in Fifteen Coastal Grapsoid Crabs Underpins Potential for Mineralization of Mangrove Production
Source: Curr Microbiol. 2024 Jun 14;81(8):224. doi: 10.1007/s00284-024-03718-5 (PMC11178586; doi:10.1007/s00284-024-03718-5)
Supplement: Supplementary file 7 — Supplementary file7 (DOCX 28 kb) [file 284_2024_3718_MOESM7_ESM.docx]

**Contribution of Aerobic Cellulolytic Gut Bacteria to Cellulose Digestion in Fifteen Coastal Grapsoid Crabs Underpins Potential for Mineralization of Mangrove Production**

*Current Microbiology*

Cheuk Yan Lee & Shing Yip Lee

Simon F.S. Li Marine Science Laboratory, School of Life Sciences, The Chinese University of Hong Kong,

Hong Kong, China

Corresponding author’s email: joesylee@cuhk.edu.hk

**Accession numbers of sequences**

**Sequence ID #Accession**

Paf1Midgut11 OP393481

Paf1Midgut12 OP393482

Paf1Midgut22 OP393483

Paf1Hindgut2 OP393484

Paf2Midgut22 OP393485

Paf2Midgut24 OP393486

Paf2Midgut25 OP393487

Paf2Midgut26 OP393488

Paf2Midgut28 OP393489

Paf2Midgut29 OP393490

Paf2Midgut30 OP393491

Paf2Midgut32 OP393492

Paf2Midgut34 OP393493

Paf2Midgut35 OP393494

Paf2Midgut36 OP393495

Paf2Midgut38 OP393496

Paf2Midgut39 OP393497

Paf2Midgut40 OP393498

Paf2Midgut43 OP393499

Paf2Stomach36 OP393500

Paf2Stomach5 OP393501

Pbi1Midgut2 OP393502

Pbi1Midgut3 OP393503

Pbi1Hindgut10 OP393504

Pbi1Hindgut11 OP393505

Pbi1Hindgut12 OP393506

Pbi1Hindgut13 OP393507

Pbi1Hindgut15 OP393508

Pbi1Hindgut2 OP393509

Pbi1Hindgut3 OP393510

Pbi1Hindgut4 OP393511

Pbi1Hindgut5 OP393512

Pbi1Hindgut7 OP393513

Pbi1Hindgut8 OP393514

Pbi1Hindgut9 OP393515

Pbi1Stomach1 OP393516

Pbi1Stomach10 OP393517

Pbi1Stomach11 OP393518

Pbi1Stomach12 OP393519

Pbi1Stomach14 OP393520

Pbi1Stomach2 OP393521

Pbi1Stomach3 OP393522

Pbi1Stomach4 OP393523

Pbi1Stomach7 OP393524

Pbi1Stomach8 OP393525

Pbi1Stomach9 OP393526

Pbi1Hindgut6 OP393527

Pbi2Midgut10 OP393528

Pbi2Midgut14 OP393529

Pbi2Midgut7 OP393530

Pbi2Stomach13 OP393531

Pbi2Stomach15 OP393532

Pbi2Stomach16 OP393533

Pbi2Stomach18 OP393534

Pbi2Stomach2 OP393535

Pbi1Hindgut14 OP393536

Cco1Stomach1 OP393537

Cco1Stomach2 OP393538

Cco1Stomach3 OP393539

Cco2Stomach.10 OP393540

Cco2Stomach1 OP393541

Cco2Stomach10 OP393542

Cco2Stomach11 OP393543

Cco2Stomach12 OP393544

Cco2Stomach3 OP393545

Cco2Stomach4 OP393546

Cco2Stomach5 OP393547

Cco2Stomach7 OP393548

Cco2Stomach8 OP393549

Cco3Stomach13 OP393550

Cco3Stomach18 OP393551

Cco3Stomach22 OP393552

Cco3Stomach25 OP393553

Cco3Stomach29 OP393554

Cco3Stomach35 OP393555

Cco3Stomach6 OP393556

Cco3Stomach8 OP393557

Cco3StomachH21 OP393558

Cco3StomachH3 OP393559

Cco3StomachH43 OP393560

Cco3StomachH44 OP393561

Cco3StomachH60 OP393562

Ode1Hindgut3 OP393563

Ode1Hindgut31 OP393564

Ode1Stomach25 OP393565

Ode3Hindgut62 OP393566

Eve1Hindgut11 OP393567

Eve1Hindgut4 OP393568

Eve1Hindgut7 OP393569

Eve1Hindgut9 OP393570

Eve2Midgut8 OP393571

Eve2Hindgut1 OP393572

Eve2Hindgut11 OP393573

Eve2Hindgut15 OP393574

Eve2Hindgut19 OP393575

Eve2Hindgut20 OP393576

Eve2Hindgut22 OP393577

Eve2Hindgut24 OP393578

Eve2Hindgut3 OP393579

Eve2Hindgut6 OP393580

Eve2Stomach11 OP393581

Eve2Stomach22 OP393582

Eve2Stomach24 OP393583

Eve2Stomach5 OP393584

Gde1Hindgut1 OP393585

Gde1Midgut2 OP393586

Gde1Midgut3 OP393587

Gde1Stomach1 OP393588

Gde1Stomach3 OP393589

Gde2Midgut2 OP393590

Gde2Midgut3 OP393591

Gde2Stomach4 OP393592

Gde2Stomach7 OP393593

Gde2Stomach8 OP393594

Cha1Stomach11 OP393595

Cha1Stomach33 OP393596

Cha2Midgut12 OP393597

Cha2Midgut39 OP393598

Cha2Hindgut11 OP393599

Cha2Hindgut18 OP393600

Cha2Hindgut20 OP393601

Cha2Hindgut35 OP393602

Cha2Hindgut38 OP393603

Cha2Hindgut41 OP393604

Cha2Hindgut43 OP393605

Cha2Hindgut5 OP393606

Cha2Hindgut55 OP393607

Cha2Hindgut57 OP393608

Cha2Stomach109 OP393609

Cha2Stomach113 OP393610

Cha2Stomach117 OP393611

Cha2Stomach29 OP393612

Cha2Stomach3 OP393613

Cha2Stomach48 OP393614

Cha2Stomach91 OP393615

Cha2Stomach95 OP393616

Cha2Stomach98 OP393617

Cha3Stomach32 OP393618

Cha4Stomach13 OP393619

Cha4Stomach5 OP393620

Hpe1Midgut1 OP393621

Hpe1Midgut2 OP393622

Hpe1Midgut3 OP393623

Hpe1Midgut4 OP393624

Hpe1Midgut5 OP393625

Hpe1Midgut6 OP393626

Hpe1Hindgut1 OP393627

Hpe1Hindgut2 OP393628

Hpe1Hindgut4 OP393629

Hpe1Hindgut5 OP393630

Hpe1Hindgut6 OP393631

Hpe1Hindgut9 OP393632

Hpe1Stomach1 OP393633

Hpe1Stomach2 OP393634

Hpe1Stomach3 OP393635

Hpe1Stomach5 OP393636

Hpe1Stomach6 OP393637

Hpe1Stomach8 OP393638

Hpe1Hindgut10 OP393639

Hpe1Hindgut7 OP393640

Hpe2Midgut1 OP393641

Hpe2Hindgut1 OP393642

Hpe2Hindgut10 OP393643

Hpe2Hindgut12 OP393644

Hpe2Hindgut13 OP393645

Hpe2Hindgut14 OP393646

Hpe2Hindgut2 OP393647

Hpe2Hindgut21 OP393648

Hpe2Hindgut3 OP393649

Hpe2Hindgut4 OP393650

Hpe2Hindgut5 OP393651

Hpe2Hindgut6 OP393652

Hpe2Hindgut7 OP393653

Hpe2Hindgut8 OP393654

Hpe2Hindgut9 OP393655

Hpe2Stomach11 OP393656

Hpe2Stomach21 OP393657

Hpe3Hindgut55 OP393658

Hpe3Stomach33 OP393659

Hpe3Stomach36 OP393660

Hpe3Stomach38 OP393661

Hpe3Stomach40 OP393662

Hpe3Stomach41 OP393663

Hpe3Stomach49 OP393664

Hpe3Stomach51 OP393665

Hpe3Stomach53 OP393666

Hpe3Stomach54 OP393667

Hpe3Stomach56 OP393668

Hpe3Stomach64 OP393669

Mfr1Hindgut1 OP393670

Mfr1Hindgut3 OP393671

Mfr1Hindgut4 OP393672

Mfr1Hindgut5 OP393673

Mfr1Hindgut6 OP393674

Mfr1Stomach1 OP393675

Mfr1Stomach3 OP393676

Mfr2Midgut3 OP393677

Mfr2Hindgut1 OP393678

Mfr2Hindgut10 OP393679

Mfr2Hindgut11 OP393680

Mfr2Hindgut12 OP393681

Mfr2Hindgut14 OP393682

Mfr2Hindgut15 OP393683

Mfr2Hindgut16 OP393684

Mfr2Hindgut17 OP393685

Mfr2Hindgut19 OP393686

Mfr2Hindgut2 OP393687

Mfr2Hindgut20 OP393688

Mfr2Hindgut21 OP393689

Mfr2Hindgut22 OP393690

Mfr2Hindgut24 OP393691

Mfr2Hindgut25 OP393692

Mfr2Hindgut26 OP393693

Mfr2Hindgut27 OP393694

Mfr2Hindgut28 OP393695

Mfr2Hindgut29 OP393696

Mfr2Hindgut3 OP393697

Mfr2Hindgut30 OP393698

Mfr2Hindgut31 OP393699

Mfr2Hindgut32 OP393700

Mfr2Hindgut4 OP393701

Mfr2Hindgut5 OP393702

Mfr2Hindgut6 OP393703

Mfr2Hindgut7 OP393704

Mfr2Hindgut8 OP393705

Mfr2Hindgut9 OP393706

Mfr2Stomach9 OP393707

Mfr3Hindgut25 OP393708

Mfr3Stomach1 OP393709

Mfr3Stomach4 OP393710

Mlo3Stomach1 OP393711

Mlo3Stomach14 OP393712

Mlo3Stomach2 OP393713

Mlo3Stomach3 OP393714

Mlo3Stomach5 OP393715

Mlo3Stomach6 OP393716

Mlo3Stomach7 OP393717

Nin1Stomach42 OP393718

Nin1Stomach8 OP393719

Nin3Midgut1 OP393720

Nin4Midgut3 OP393721

Nin3Hindgut1 OP393722

Nin4Hindgut10 OP393723

Nin3Hindgut2 OP393724

Nin3Hindgut3 OP393725

Nin3Hindgut4 OP393726

Nin3Hindgut6 OP393727

Nin4Hindgut8 OP393728

Nin3Stomach22 OP393729

Nin3Stomach23 OP393730

Nin3Stomach30 OP393731

Ppi2Hindgut3 OP393732

Ppi1Hindgut1 OP393733

Ppi1Hindgut10 OP393734

Ppi1Hindgut11 OP393735

Ppi1Hindgut12 OP393736

Ppi1Hindgut13 OP393737

Ppi1Hindgut2 OP393738

Ppi1Hindgut3 OP393739

Ppi1Hindgut4 OP393740

Ppi1Hindgut5 OP393741

Ppi1Hindgut7 OP393742

Ppi1Hindgut8 OP393743

Ppi1Stomach4 OP393744

Ppi1Stomach5 OP393745

Ppi1Stomach6 OP393746

Ppi2Hindgut1 OP393747

Ppi2Hindgut10 OP393748

Ppi2Hindgut11 OP393749

Ppi2Hindgut13 OP393750

Ppi2Hindgut14 OP393751

Ppi2Hindgut15 OP393752

Ppi2Hindgut16 OP393753

Ppi2Hindgut2 OP393754

Ppi2Hindgut4 OP393755

Ppi2Hindgut5 OP393756

Ppi2Hindgut6 OP393757

Ppi2Hindgut7 OP393758

Ppi2Hindgut8 OP393759

Ppi2Hindgut9 OP393760

Ppi2Stomach2 OP393761

Ppi2Stomach6 OP393762

Opa1Hindgut27 OP393763

Opa1Stomach10 OP393764

Opa1Stomach11 OP393765

Opa1Stomach12 OP393766

Opa1Stomach13 OP393767

Opa1Stomach5 OP393768

Opa1Stomach6 OP393769

Opa1Stomach7 OP393770

Opa1Stomach8 OP393771

Opa2Stomach12 OP393772

Opa2Stomach25 OP393773

Opa2Stomach29 OP393774

Opa3Stomach3 OP393775

Opa3Stomach4 OP393776

Opa3Stomach46 OP393777

Opa3Stomach56 OP393778

Opa3Stomach26 OP393779

Opa3Stomach29 OP393780

Opa3Stomach34 OP393781

Opa5Stomach10 OP393782

Cco1Midgut1 OP393783

Cco1Midgut2 OP393784

Cco1Midgut3 OP393785

Cco1Midgut4 OP393786

Cco1Midgut5 OP393787

Cco1Midgut6 OP393788

Cco1Midgut7 OP393789

Cco1Hindgut1 OP393790

Cco1Hindgut2 OP393791

Cco1Hindgut3 OP393792

Cco1Hindgut4 OP393793

Cco1Hindgut5 OP393794

Cco1Hindgut7 OP393795

Cco1Hindgut6 OP393796

Oin1Stomach12 OP393797

Oin1Stomach13 OP393798

Oin1Stomach3 OP393799

Oin1Stomach41 OP393800

Oin1Stomach43 OP393801

Oin1Stomach50 OP393802

Oin1Stomach53 OP393803

Oin1Stomach54 OP393804

Oin1Stomach55 OP393805

Oin1Stomach64 OP393806

Oin1Stomach70 OP393807

Oin1Stomach9 OP393808

Oin1Stomach11 OP393809

Oin1Stomach21 OP393810

Oin1Stomach24 OP393811

Oin1Stomach25 OP393812

Oin1Stomach27 OP393813

Oin1Stomach30 OP393814

Oin1Stomach32 OP393815

Oin1Stomach34 OP393816

Oin1Stomach35 OP393817

Oin1Stomach4 OP393818

Oin1Stomach5 OP393819

Oin1Stomach51 OP393820

Oin1Stomach7 OP393821

Oin1Stomach71 OP393822

Oin1Stomach8 OP393823

Oin2Hindgut31 OP393824

Oin2Hindgut38 OP393825

Oin2Hindgut39 OP393826

Oin2Hindgut44 OP393827

Oin2Hindgut51 OP393828

Oin2Hindgut57 OP393829

Oin2Stomach7 OP393830

Oin3Hindgut1 OP393831

Vyu1Hindgut1 OP393832

Vyu2Midgut1 OP393833

Vyu2Stomach4 OP393834

Vyu2Stomach9 OP393835

Vyu3Hindgut10 OP393836

Vyu3Hindgut2 OP393837

Vyu3Hindgut4 OP393838

Vyu3Hindgut6 OP393839

Vyu3Stomach1 OP393840

Vyu3Stomach11 OP393841

Vyu3Stomach14 OP393842

Vyu3Stomach15 OP393843

Vyu3Stomach16 OP393844

Vyu3Stomach18 OP393845

Vyu3Stomach2 OP393846

Vyu3Stomach21 OP393847

Vyu3Stomach23 OP393848

Vyu3Stomach24 OP393849

Vyu3Stomach25 OP393850

Vyu3Stomach26 OP393851

Vyu3Stomach27 OP393852

Vyu3Stomach28 OP393853

Vyu3Stomach29 OP393854

Vyu3Stomach3 OP393855

Vyu3Stomach31 OP393856

Vyu3Stomach33 OP393857

Vyu3Stomach34 OP393858

Vyu3Stomach35 OP393859

Vyu3Stomach36 OP393860

Vyu3Stomach37 OP393861

Vyu3Stomach41 OP393862

Vyu3Stomach42 OP393863

Vyu3Stomach50 OP393864

Vyu3Stomach7 OP393865

Vyu3Stomach8 OP393866

**Table S1. Substitution model used to build phylogenetic trees.**

|  | Substitution model |
| --- | --- |
| *Bacillus* | GTR+F+I+G4 |
| *Vibrio* | GTR+F+I+G4 |
| *Klebsiella* | HKY+F+I+G4 |
| *Mangrovibacter,* | HKY+F+G4 |
| *Novosphingobium* | HKY+F+I+G4 |
| *Microbacterium* | GTR+F+I+G4 |
| *Pseudocitrobacter* | HKY+F+G4 |
